# Building Exposure Biology Centers to Put the E into “G × E” Interaction Studies

**DOI:** 10.1289/ehp.12812

**Published:** 2009-08

**Authors:** Martyn T. Smith, Stephen M. Rappaport

**Affiliations:** School of Public Health, University of California, Berkeley, California, E-mail: martynts@berkeley.edu, srappaport@berkeley.edu

Adverse gene–environment interactions (G × E) probably influence most chronic diseases, including neurologic disorders and cancer. The genetic (G) contribution to different diseases varies, but several lines of evidence clearly show that nongenetic factors have high attributable risks, often in the range of 80–90% (Willett 2002). The dominance of nongenetic components highlights the importance of the environment (E) to chronic disease risks.

Genomic tools arising from the Human Genome Project (HGP), combined with bioinformatics studies, have allowed epidemiologists to examine the genetic component of chronic diseases. Genome-wide association studies offer glimpses of the roles that particular genes play in disease development. However, the genetic factors identified thus far have generally been of low penetrance (a few percent at most) and have mainly offered clues as to which G × E (and G × G) effects might be worth pursuing.

In contrast, the tools for quantitative assessment of exposures—based on measurements of chemicals in air, water, food, and the human body—have changed little since the 1970s. The lack of high-throughput methods of exposure assessment has motivated epidemiologists to rely upon self-reported data to categorize chemical exposures from environmental, endogenous, and dietary sources. With the possible exceptions of smoking and alcohol consumption, such self-reports have been unreliable predictors of long-term exposure levels and are poorly suited for detecting G × E effects.

Although 30 years of investment in G now illuminates genetic determinants of diseases, we are still in the dark ages when it comes to quantifying E (i.e., human exposures). Recognizing the disparity in current knowledge between genes and environmental exposures, Wild (2005) defined the “exposome,” representing all environmental exposures (including those from diet, lifestyle, and endogenous sources) from conception onward, as a quantity of critical interest to disease etiology. If we expect to have any success at identifying the effects of E, G, and G × E on chronic diseases, we must develop 21st-century tools to measure exposure levels in human populations. That is, we need an HGP-like commitment to quantify the exposome.

Elaborating the exposome will be challenging, but some promising analytical approaches are emerging from microfluidics, nano-technologies, and mass spectrometry (MS). For example, a capillary lab-on-a-chip system has been developed to detect polycyclic aromatic hydrocarbons at parts-per-billion levels on the surface of Mars (Stockton et al. 2009), and surface enhanced Raman spectroscopy using silver nanoparticles has detected (and speciated) arsenic at parts-per-billion levels in water samples (Mulvihill et al. 2008). Both of these devices are portable, capable of high throughput, and can be adapted to other contaminants of interest.

Technologic developments with liquid chromatography/tandem MS (LC-MS/MS) now motivate ultrasensitive measurements of protein adducts; these are excellent long-term biomarkers of exposure and internal dose for carcinogens (Rubino et al. 2009). Indeed, with National Institutes of Health Genes and Environment Initiative funding, we are using protein adductomics to profile human exposures in archived serum from cancer case–control studies. We ultimately envision an analytical platform to rapidly quantify protein adducts in much the same way that the DNA sequencer made possible the success of the HGP. Given the relentless improvements in MS sensitivity, it is realistic to expect that this technology will be applied with a single drop of blood. In fact, our recent measurement of a benzene-related adduct in dried blood spots opens the door to measurements of *in utero* chemical exposures, using archived neo natal blood spots (Funk et al. 2008).

Simple and inexpensive monitoring methods can motivate reductions in exposures to toxic chemicals, as has been observed with inorganic lead (Pirkle et al. 2005). Indeed, given people’s concerns about elevated levels of xeno biotic chemicals in their bodies, commercial markets could ultimately develop for exposure sensors. However, without a regulatory mandate, we cannot rely upon the free market alone to generate an exposure-sensing industry. We must focus instead upon the profound shortcomings that epidemiologists face in discovering environmental causes of chronic diseases without adequate exposure data. Facing a similar dilemma three decades ago, the HGP created several DNA sequencing centers to rapidly sequence the genome, and thereby created an infrastructure from which we still benefit. Imagine if we could build six or seven exposure biology centers to quantify chemical exposures rapidly and at low cost. The impact such centers would have on our understanding of E and G × E as determinants of human disease would be extraordinary.

True, an HGP-like effort for the environment would require a large investment. However, at a government cost of $2.7 billion (in $1991), HGP technologies will generate a projected $45 billion this year in sales. It does not require a great leap of faith to expect a similar multiplier from concerted action to quantify human exposures. As important sources of exposure are recognized and controlled, one can also anticipate reductions in morbidity and mortality that would translate into enormous savings in health care expenses.

## Figures and Tables

**Figure:**
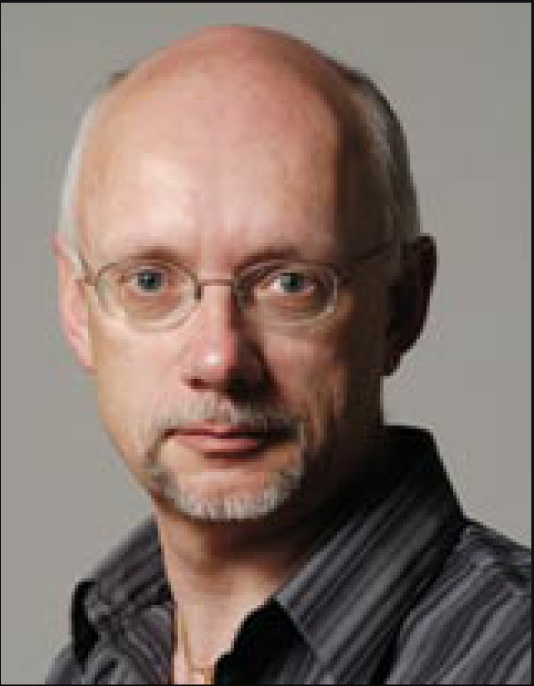
Martyn T. Smith

**Figure:**
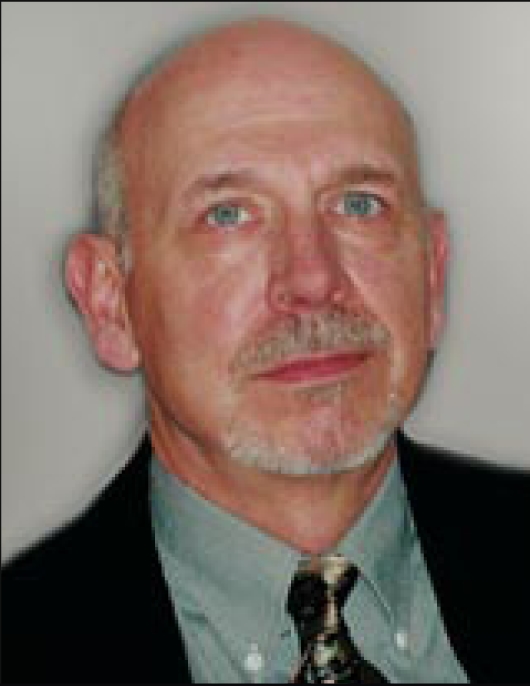
Stephen M. Rappaport
